# Discrete choice modelling of hypertension patients’ preferences for attributes of a public medical facility in Ibadan, Nigeria

**DOI:** 10.1186/s12913-025-12257-z

**Published:** 2025-01-16

**Authors:** Oluwaseun Aramide Otekunrin, Tekiyat Abiola Adebisi, Sururoh Adeniran-Babatunde, Olutosin Ademola Otekunrin

**Affiliations:** 1 Statistical Design of Investigations Unit, Department of Statistics, Faculty of Science, University of Ibadan, Ibadan, Nigeria; 2 Department of Obstetrics and Gynaecology, Federal Teaching Hospital, Katsina, Nigeria; 3 Shrewbury and Telford NHS Trust, Mytton Oak Rd, Shrewbury, SY3 8XQ UK; 4 Innovation Lab for Policy Leadership in Agriculture and Food Security (PiLAF), University of Ibadan, Ibadan, Nigeria

**Keywords:** Hypertension patients’ preferences, Discrete choice experiments, Public medical facility, Mixed logit model, Nigeria

## Abstract

**Background:**

Not much is known about hypertension patients’ preferences for attributes of public medical facilities in Nigeria and how these preferences influence their choices of medical facilities for treatment. An understanding of what these patients want especially in terms of service delivery could contribute to improved hypertension control.

**Objective:**

This study aimed to determine hypertension patients’ preferences for attributes of a public medical facility in Ibadan, Nigeria.

**Methods:**

A Discrete Choice Experiment (DCE) that utilized three hypothetical medical facilities was used for the study. A simple random sample (SRS) of 150 eligible hypertension patients was selected from a secondary medical facility in Ibadan, Nigeria. An efficient D-optimal choice design was adopted and used in generating nine hypothetical choice sets for the experiment. Each patient was expected to study the nine sets carefully and choose an option from each of the sets. The first choice set was repeated as the tenth set to examine respondents’ consistency. The DCE questionnaires were administered using a one-to-one interview method. A mixed logit regression modeling technique was used to obtain parameter and Willingness to Pay (WTP) estimates.

**Results:**

The patients preferred medical facilities with waiting time before consultation with medical doctors to be between thirty minutes and one hour. The attribute level ‘‘a lot of information’’ was the most preferred. The patients were unwilling to pay for the “little or no drugs and diagnostic equipment” attribute level. A negative and significant coefficient for cost indicated that higher out-of-pocket costs negatively affected hypertension patients’ choice of a public medical facility.

**Conclusion:**

Hypertension patients attending a public medical facility in Ibadan, Nigeria preferred a facility with access to comprehensive information about their health in addition to reasonable waiting times, availability of a lot of drugs and diagnostic equipment, and reduced out-of-pocket costs. Provision of healthcare services that align with these preferences could enhance patient satisfaction thus contributing to improved hypertension control.

**Supplementary Information:**

The online version contains supplementary material available at 10.1186/s12913-025-12257-z.

## Introduction

Hypertension is a serious non-communicable disease in which blood vessels have constantly raised pressure [1, 2]. It is one of the most leading cause of death among non-communicable diseases, and contributes significantly to stroke, coronary heart diseases and renal complications [3, 4]. Globally, it affects an estimated 33% of adults aged 30–79 years with the population of people living with hypertension increasing from 650 million in 1990 to 1.3 billion worldwide in 2019 [4, 5]. The disease is a major public health issue in Nigeria with an estimated 1 in 3 persons having hypertension and 25% of emergency admissions in urban hospitals being the result of hypertension-related complications [6]. To reverse this ugly trend, the Federal Ministry of Health in collaboration with the National Primary Health Care Development Agency launched a pilot program of the National Hypertension Control Initiative (NHCI) in 2020, with the support of the World Health Organization (WHO). The NHCI is aimed at expanding blood pressure screening and promoting early detection and treatment of uncomplicated hypertension in Nigeria’s Primary health care Centers (PHCs). The PHCs are closer to rural and underserved communities where access to healthcare is generally poor. The initiative has successfully improved early detection of elevated blood pressure, especially in unsuspecting patients, while the number of diagnosed patients has grown by over 50% in the second year of the program. Despite these successes, the hypertension control rate in Nigeria is 11% [4]. This low rate has been attributed to many factors including financial constraints on the part of the patients and unsatisfactory services rendered by public medical facilities, among others [7]. An understanding of what these patients want especially in terms of service delivery could contribute to improved hypertension control. But, not much is known about hypertension patients’ preferences for attributes of public medical facilities in Nigeria and how these preferences influence their choices of public medical facilities for treatment. Therefore, this study was aimed at determining factors hypertension patients’ consider before choosing a public medical facility for treatment. Recent researches have focused on the economic burden and medication adherence among hypertension patients in Nigeria, and management of hypertension at public primary healthcare centers in Nigeria [8–12]. Also, a study to determine whether patient satisfaction influences medication adherence and blood pressure control among hypertension patients was conducted at Federal Medical Centre, Asaba, Delta State, Nigeria [13]. Discrete Choice Experiment (DCE) is a survey method used to understand how individuals make decisions and to quantify the relative importance of features of such experiments [14]. Understanding what patients want is an important aspect of patient-centered care and DCEs are increasingly being used by policymakers to achieve this important goal [15]. A DCE was used to identify hypertension patients’ preferences for healthcare services in China [16]. Also, a DCE was used to explore patient preferences for the management of hypertension in the UK [17]. Further, a DCE was used to determine patient preferences for pharmaceutical and device-based treatments for uncontrolled hypertension in the USA [14]. Therefore, in this paper, we examine hypertension patients’ preferences for public medical facilities in Ibadan, Nigeria using a DCE. These results would assist relevant policymakers to take evidence-based decisions that would lead to better service delivery for hypertension patients in our public medical facilities which would ultimately lead to improved hypertension control in Ibadan, Nigeria.

## Methods

### Study location

Ibadan, the capital of Oyo State, is located in southwest, Nigeria. It is one of the most densely populated cities in Nigeria, with an estimated population of 4,004,000 [18]. The DCE was conducted at a purposively selected secondary medical facility in the city. The medical facility is one of the largest state-owned hospitals in Oyo State. It is also one of the few state-owned hospitals that run Medical Out-Patient (MOP) clinics. A specialist hypertension clinic, where hypertensive patients are treated and followed-up, runs twice a week at the hospital with at least 30 patients attended to each clinic day. This hospital was selected because most patients choose the hospitals which they consider give 'best service', rather than the ones nearest to them [19]. Further, many of these patients do not go to primary healthcare facilities that are closer to their places of residence because these primary healthcare facilities are mostly ill-equipped and poorly managed by the government [12].

### Discrete choice experiment (DCE)

A DCE is a quantitative technique used to estimate the relative importance consumers assign to the attributes of a product or service rendered coupled with their trade-offs against one another [20–22]. A DCE consists of a set of hypothetical choice sets made up of attributes with at least two levels. Each choice set consists of two or more options (or alternatives). Each respondent is shown each choice set in turn and asked to choose one of the options presented in the choice set. The number of options in a choice set is called the choice set size [22–25].

DCEs have been found useful in different fields of human endeavor including the healthcare sector. The relative importance of attributes that affect choice of under-five child healthcare services by caregivers was examined using a DCE [26]. They found out that caregivers' strongly valued healthcare services where adequate medical equipment and medicines are readily available. Attributes women consider as most important before choosing a long-acting reversible contraceptive have also been identified via a DCE [22].

## Experimental design of the DCE

### Identification of attributes and levels

A comprehensive literature search was conducted and this led to the identification of general themes for the study [27–30]. The themes identified are speed of access, expert advice, affordability, and infrastructure, equipment and drugs. A focus group discussion, which lasted for about 50 min, was conducted with seven experienced general medical practitioners who work in public medical facilities located in Ibadan, but which were not selected for the survey. Relevant questions on each theme were asked and these generated robust discussion among the doctors. These doctors were selected because of the experience they have gathered, over time, interacting with and managing patients from diverse socio-economic backgrounds within and outside the study area. The medical doctors assisted in identifying the attributes that were relevant and the range of levels that were feasible for each attribute in the local context. Furthermore, these attributes were compared with those found in related literature and main attributes common to the two sources were finally selected for the study. These are waiting time before consultation, information transfer from doctor to patient, availability of drugs and diagnostic equipment, and out-of-pocket costs per clinic visit. Each of these attributes has three levels represented as 0, 1 and 2, respectively giving a total of 81 ${(3}^{4})$ treatment combinations. The attributes and levels, as well as an illustration of a choice set are presented in Tables 1 and 2, respectively.

**Table 1:** Attributes and levels

*Attributes*	*Levels*	*Description*
***Waiting time before consultation***	0	< 30 min
	1	30 min – 1 h
	2	> 1 h
***Information transfer from doctor to patient***	0	Little or no information
	1	Some information
	2	A lot of information
***Availability of drugs and diagnostic equipment***	0	Little or no drugs and diagnostic equipment
	1	Some drugs and diagnostic equipment
	2	A lot of drugs and diagnostic equipment
***Out-of-pocket costs per visit***	0	N7000 NGN
	1	N10,000 NGN
	2	N13,000 NGN

1USD = N788.50 NGN, Nov.1, 2023

**Table 2:**
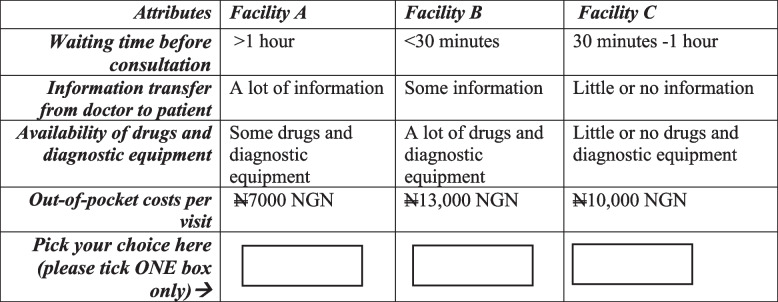
Illustration of a choice set

To reduce the size of the experiment, an efficient D-optimal choice design was adopted and used in generating nine hypothetical choice sets for the DCE [22, 31, 32]. Each choice set had three options and each option comprised levels of the four different attributes. Each participant is expected to study the nine sets carefully and choose an option from each of the sets. It is assumed that respondents will consider all the attribute levels and make rational choices. The first choice set was repeated as the tenth set to examine respondents’ consistency.

Since a choice set can consist of two or more options (or alternatives), the use of three alternatives would not lead to a cognitive burden for the respondents. Additionally, cognitive burden usually becomes a challenge with an increasing number of attributes and levels. In this study, the number of attributes (4) and levels (3 for each attribute) considered is not excessive, so cognitive burden would not be a challenge for the respondents. Furthermore, the opt-out alternative was not considered because all combinations were plausible. The choice design is efficient for the estimation of main effects in the multinomial logit model and other models especially those that accommodate preference heterogeneity [31, 32]. The DCE questionnaire is attached (Supplementary file).

The face validity was measured by setting a priori hypotheses about the attribute levels and these were confirmed by examining the direction of the preference estimates. The following hypotheses were tested:

- (1) hypertension patients prefer a public medical facility where the waiting time before consultation is not more than an hour.
- (2) patients value the transfer of a lot of health-related information from their doctors more than any other attribute of a public medical facility.
- (3) patients prefer a public medical facility equipped with a lot of drugs and diagnostic equipment.

Pilot-testing of the questionnaire among 15 eligible patients who were not part of the target population was done and necessary changes were made on the questionnaire to remove all forms of ambiguity. Overall assessment of the questionnaire was finally conducted before the administration of the questionnaire.

### Sample size determination

A rule of the thumb,

$$\frac{nta}{c}\ge 500$$

where $n$ is the number of respondents, $t$ is the number of choice sets, $a$ is the number of alternatives, and $c$ is the largest number of levels for any of the attributes, was used for sample size determination [33]. This resulted in $n=56$ for this study, but was increased to 150 to satisfy reliable model estimation rules [22, 34].

#### Inclusion criteria

Hypertension patients aged at least 18 years on regular follow-up treatment, with or without blood pressure control, at the MOP clinic of the medical facility and have been diagnosed with hypertension for at least six months.

#### Exclusion criteria

Patients who came to the clinic for the first time, pregnant, and very ill hypertensive patients were excluded from the study.

### Data collection and analysis

The interviewers received training on the objective of the research and their responsibilities during the administration of the questionnaire. The questionnaire was divided into three sub-sections. The first section presented the aim of the research and how to fill the questionnaire. Information on socio-demographic and clinical profiles of the respondents were in section two, while section three contained the choice sets. The questionnaires were administered through individual, face-to-face interviews to ensure that they were completed accurately and returned, thereby increasing the survey’s reliability. The survey was conducted in English Language but translation to the local Yoruba language was done for respondents with no formal education (4%). The DCE started on July 13, 2023 and was completed on August 31, 2023. Each clinic day, a list of patients who satisfied the inclusion criteria was obtained from the hospital staff in charge of the patients’ records and this list constituted the sampling frame. A Simple Random Sampling (SRS) procedure was used to select 10 patients each from the sampling frame every clinic day. This is presented in Table 3.

**Table 3:** Sampling procedure

Clinic days	Number of eligible patients	SRS from eligible patients
**1**	33	10
**2**	30	10
**3**	32	10
**4**	32	10
**5**	34	10
**6**	34	10
**7**	30	10
**8**	30	10
**9**	32	10
**10**	30	10
**11**	32	10
**12**	30	10
**13**	31	10
**14**	31	10
**15**	30	10
Total	**471**	**150**

Each patient used at least 20 min to complete the DCE questionnaire.

Statistical analysis was conducted using STATA 14. Descriptive statistics on the socio-demographic and clinical profiles of the respondents were obtained. The influence of selected attributes on hypertension patients’ choice of medical facility was estimated using the mixed logit model [22, 35].

The mixed logit model was selected because of its ability to overcome the limitations of the traditional multinomial logit model which are the assumptions of independence of observations and equal preferences for the respondents [22, 25, 36]. Given that $\beta$ varies randomly across individual respondents, $f\left(\beta /\delta \right)$ is a mixture of continuous parametric distributions and $\delta$ refers to parameters of that mixture distribution, then

1 $${P}_{jns}\left({y}_{j}=1\right)={\int }_{{\beta }_{sjk}}\left[\frac{exp\left({\beta }_{sjk}{X}_{jk}\right)}{\sum_{j=1}^{J}exp\left({\beta }_{sjk}{X}_{jk}\right)}\right]f\left(\beta /\delta \right)\partial {\beta }_{sjk}$$

where Eq. (1) is the choice probability for alternative product $j$ (out of all $J$ products) and the values of ${\beta }_{sjk}$ are drawn from the continuous mixture distribution $f\left(\beta /\delta \right)$ [35].

Willingness-to-pay (WTP) is the marginal rate of substitution between the non-monetary attribute and the monetary/cost attribute, assuming only one product is available and that it is chosen with a 100% certainty. It is computed by dividing the coefficient of the non-monetary attribute with the coefficient of the cost attribute $\left(-1\left(\frac{\widehat{{\beta }_{attribute}}}{\widehat{{\beta }_{cost}}}\right)\right)$ [35, 37]. The WTP estimates and their confidence intervals were calculated using the approach described in [37].

## Results

A 100% response rate was recorded. Preference estimates had expected signs in line with a priori hypotheses and expectations, thus establishing the face validity of the model.

### Patients’ profiles

The mean age of the respondents was 53.6 years and majority of them were females (78%). More than half (62%) of them were in the 40–59 age group. More than half (67.3%) were working. The respondents’ mean monthly income was N60,463 NGN ($76.68 USD) while 55.3% earned between N30,000 NGN ($38.05 USD) and N60,000 NGN ($76.09 USD) per month. Four percent had no formal education. More than half (57.3%) of the respondents did not have health insurance policy while 47.3% reported a family history of hypertension. About 13.3% of them have been managing the disease for at least nine years while 33.3% had comorbidities (Tables 4 & 5).

**Table 4:** Socio-demographic profile of the respondents

*Variable*	*Frequency (n)*	*Percent (%)*
***Gender***		
*Female*	117	78.0
*Male*	33	22.0
***Age***		
*20–39*	14	9.3
*40–59*	93	62.0
*60 and above*	43	28.7
***Marital status***		
*Married/Cohabiting*	81	54.0
*Single/Separated/Widowed*	69	46.0
***Education***		
*No formal education*	6	4.0
*Secondary education or less*	92	61.3
*Tertiary education*	52	34.7
***Income (per month)***		
*Less than N30,000*	21	14.0
*N 30,000 – N 60,000*	83	55.3
*Above N 60,000*	46	30.7
***Religion***		
*Christianity*	57	38.0
*Islam*	93	62.0
***Employment***		
*Employed*	101	67.3
*Unemployed*	49	32.7
***Family type***		
*Monogamous*	82	54.7
*Polygamous*	68	45.3
***Household size***		
*1 – 4*	67	44.7
*5 and above*	83	55.3

1 USD = N788.50 NGN, Nov.1, 2023

**Table 5:** Clinical profile of the respondents

*Variable*	*Frequency (n)*	*Percent (%)*
***Health insurance policy***		
*NHIS*	12	8.0
*None*	86	57.3
*OYHIS*	52	34.7
***Comorbidities***		
*No*	100	66.7
*Yes*	50	33.3
***Self-reported health status***		
*Poor*	8	5.3
*Fair*	91	60.7
*Very Good*	51	34.0
***Duration of diagnosis ( years)***		
*1—4*	71	47.3
*5—8*	59	39.3
*9 and above*	20	13.3
***Family history of disease***		
*No*	79	52.7
*Yes*	71	47.3

### Hypertension patients’ preferences for attributes of a public medical facility

The coefficient of ‘ < 30 min’ is positive but not statistically significant at 5% level of significance. However, the coefficient of ‘30 min—1 h’ is positive and significant.. This shows that, averagely, the patients desired a public medical facility that possesses this characteristic more than a facility that possessed ‘ > 1 h’ characteristic.

The coefficients of ‘some information’ and ‘a lot of information’ are both positive and significant. However, the attribute level ‘a lot of information’ had the highest influence on the probability of choosing a medical facility (*P* < 0.001) implying that it is the most important characteristic of all the attribute levels. Thus on average, the patients prefer public medical facility where information dissemination from the doctor to patients is prioritized to public medical facilities where this attribute level is lacking.

Furthermore, the coefficient of ‘a lot of drugs and diagnostic equipment’ is positive and significant indicating that on average, the patients prefer a public medical facility equipped with a lot of drugs and diagnostic equipment to medical facilities that have some drugs and diagnostic equipment. In addition, a negative and significant coefficient for ‘little or no drugs and diagnostic equipment’ implies a strong aversion for a public medical facility that possess this characteristic.

The coefficient of cost is negative and significant indicating strong aversion for very high out-of-pocket costs. The mixed logit model estimates are in Table 6.

**Table 6:** Estimates of hypertension patients’ preferences for attributes of public medical facilities

*Variable*	*Coefficient*	*Standard Error*	*P* >*|z|*
*Cost*	−0.000	0.000	0.000
*Standard Deviation*	0.001	0.000	0.000
< *30 min*	0.265	0.141	0.059
*Standard Deviation*	0.143	0.146	0.328
*30 min—1 h*	0.246	0.122	0.044
*Standard Deviation*	−0.008	0.124	0.952
*Some information*	1.976	0.147	0.000
*Standard Deviation*	−0.620	0.199	0.002
*A lot of information*	2.352	0.185	0.000
*Standard Deviation*	−1.425	0.184	0.000
*Little or no drugs and diagnostic equipment*	−2.579	0.262	0.000
*Standard Deviation*	2.013	0.256	0.000
*A lot of drugs and diagnostic equipment*	0.757	0.118	0.000
*Standard Deviation*	0.837	0.155	0.000

Number of observation = 150; log likelihood = −904.1213, likelihood ratio ${{\varvec{\chi}}}^{2}$(7) = 347.44, Probability > ${{\varvec{\chi}}}^{2}$ = 0.0000

### Hypertension patients’ WTP for attributes of a public medical facility

Results of the mean WTP are presented in Table 7. The significant attribute levels are ‘between 30 min and 1 h’, ‘some information’, ‘a lot of information’, ‘little or no drugs and diagnostic equipment’ and ‘a lot of drugs and diagnostic equipment’. The mean WTP for ‘little or no drugs and diagnostic equipment’ is negative (- N8712.562 NGN [-USD 11.05]) indicating patients’ strong aversion for the level.

**Table 7:** Hypertension patients’ WTP for attributes of public medical facilities

***Variables***	***WTP Estimates* (**N)	***95% confidence interval***
***Waiting time before consultation***		
< *30 min*	895.933	(−18.501, 1810.367)
*30 min—1 h*	831.298	(23.276, 1639.320)*
***Information transfer from doctor to patient***		
*Some information*	6674.194	(4796.232, 8552.157)*
*A lot of information*	7943.985	(5600.492, 10,287.479)*
***Availability of drugs and diagnostic equipment***		
*Little or no drugs and diagnostic equipment*	−8712.562	(−11,511.805, −5913.320)*
*A lot of drugs and diagnostic equipment*	2555.579	(1528.994, 3582.164)*

WTP estimates are based on Table 5, *confidence interval does not include zero

## Discussion

This study aimed to determine hypertension patients’ preferences for attributes of a public medical facility and their willingness to pay for such attributes. More than half (62%) of the patients are in the 40–59 age group. Using the American College of Cardiology/American Heart Association (ACC/AHA) 2017 guidelines, a higher prevalence of hypertension (71.3%) among adults of the same age group in Lagos, Nigeria was reported [38].

More than half (57.3%) of the patients are not enrolled into either the state or national health insurance scheme. Also, a study reported poor health insurance coverage from Nigeria [39]. The patients prefer a public medical facility where the waiting time before consultation is between 30 min and 1 h. They are willing to pay an extra N831.298 NGN ($1.05 USD) to get this attribute level in their chosen public medical facility. In a study conducted in China in 2021, [16] reported that the waiting time were positive predictors of hypertension patients’ choice of healthcare services.

Notably, this study has been able to show that hypertension patients in our study area value a lot of health-related information from medical practitioners. They are willing to pay an extra N7943.9851 NGN ($10.07 USD, as at Nov.1, 2023) to get a lot of health-related information from medical practitioners. But, not all these respondents would be able to pay an extra N16, 114.82 ($10.07 USD) as at July 13, 2024 because the current economic hardship in Nigeria has affected the source of livelihood of most Nigerians making access to basic needs, including health, difficult. However, a study conducted in Kano, a city in Northern Nigeria showed that 75.2% of doctors who participated in the study did not value sharing much information with their patients [40].

Hypertensive patients value a medical facility equipped with a lot of drugs and diagnostic equipment. They are willing to pay an extra N2, 555.579 ($3.24 USD, as at Nov.1, 2023) for this attribute level. This is further emphasized by the patients’ unwillingness to pay for the “little or no drugs and diagnostic equipment” attribute level (significant negative mean WTP result). However, in a study on public preferences for primary care models, [41] reported that respondents preferred a primary care centre with many diagnostic facilities.

Negative and significant coefficient for cost are as expected a priori, implying that higher out-of-pocket costs incurred in accessing public medical facilities negatively affect the choice of a public medical facility. The patients preferred very low out-of-pocket costs. Harsh economic realities in Nigeria would negatively affect regular clinic attendance which could impact negatively on adherence to medications. Furthermore, the cost of medication is a major hindrance to effective treatment of hypertension [42]. According to Ipinnimo et al., [1], the average monthly cost of care of hypertension in Ekiti State, Southwest Nigeria was ₦15,964.76 NGN ($44.35 USD), while medication costs in sub-Saharan Africa ranged between $1.70 and $97.06 USD [43].

Results from this study show that patients are willing to pay more for a lot of health related information from medical practitioners which suggests the desire to be actively engaged in their care. These findings provide evidence that emphasizes the need for the government to embrace a patient-centered approach, rather than the traditional paternalistic approach, in the provision and management of public healthcare facilities in Nigeria.

Relevant authorities should endeavor to provide healthcare services that align with what hypertension patients want so as to enhance service delivery and patient satisfaction, thus contributing to regular clinic attendance and improved hypertension control.

## Limitations of this study

The study was conducted in a secondary medical facility in Ibadan. Thus, results obtained might not be representative of the entire hypertension patients in Nigeria. Future studies should capture hypertension patients’ preferences in all geo-political zones of Nigeria. Secondly, this study did not incorporate preferences of hypertension patients who utilize private medical facilities. This group should be included in future studies. Thirdly, the hypothetical nature of the experiment made external validation difficult. Qualitative studies should be part of similar experiments in future so that external validity can be measured. Furthermore, males were underrepresented in the survey (22%). Quota sampling should be considered in the future to improve generalizability.

## Conclusion

In this paper, we determined hypertension patients’ preferences for attributes of a secondary public medical facility and their willingness to pay for such attributes in Ibadan, southwest Nigeria. Hypertension patients prefer a public medical facility where the total out-of-pocket costs is very low and waiting time before consultation range between 30 min and 1 h. They also value a lot of health-related information from medical practitioners, they are willing to pay extra N7943.9851 NGN ($10.07 USD) to get this attribute level. Furthermore, they prefer a facility equipped with a lot of drugs and diagnostic equipment. These results would assist relevant policymakers to make informed decisions that would lead to better service delivery in the public medical facility in Ibadan, Nigeria.

## Supplementary Information

Supplementary Material 1.

## Data Availability

Data sets generated during the current study are available from the corresponding author on reasonable request.
