# Supplementary material for: Discrete choice modelling of hypertension patients’ preferences for attributes of a public medical facility in Ibadan, Nigeria
Source: BMC Health Serv Res. 2025 Jan 16;25:92. doi: 10.1186/s12913-025-12257-z (PMC11740352; doi:10.1186/s12913-025-12257-z)
Supplement: Supplementary file 1 — Supplementary Material 1. [file 12913_2025_12257_MOESM1_ESM.docx]

**Discrete Choice Experiment (DCE) Questionnaire**

**Informed Consent Form**

This DCE questionnaire seeks to collect information from hypertension patients attending public Medical Out-Patients (MOP) clinic at the Ring Road State Hospital, Challenge, Ibadan. The information is on preferences for attributes of public health facilities. All of the provided answers will be kept confidential. No identifying information will be publicly provided. The data will be summarily reported and individual responses will be anonymously treated. The questionnaire should not take more than 20 minutes to complete.

Your participation in this research is entirely voluntary. If you choose to participate, kindly indicate your consent by signing below:

Signature of Respondent:………………… Date:…………………….

Thank you for agreeing to be a part of the survey.

Kindly tick (✓) as appropriate

**Section A: Socio-Demographic Information**

1. Gender: Male ( ) Female ( )

2. Age (in years): ……… 3. Household size……………

4. Employment status: Employed ( ) Unemployed ( ) 5. Monthly income (#): ………………….

6. Highest Educational Qualification: None ( ) Secondary education or less ( ) Tertiary education ( )

7. Religion: Christianity ( ) Islam ( ) Traditional ( ) Others ( )

8. Marital status: Single/separated ( ) Married/cohabiting ( ) Widowed ( )

9. Family type: Monogamous ( ) Polygamous ( ) 10. Family history of hypertension: Yes ( ) No ( )

11. Health insurance policy: None ( ) NHIS ( ) OYHIS ( ) Others, please specify………………………..

12. Number of years you have been managing hypertension: ….. 13. Comorbidities: Yes ( ) No ( )

14. Health Status: Poor ( ) Fair ( ) Very good ( )

**Section B: Choice Sets**

This section contains ten imaginary scenarios (**Choice Set 1 – 10**) that describes different attributes of public health facilities in Nigeria. Kindly think about each scenario as if you were deciding among them in the real world. Please select your preferred option either from **FACILITY A or FACILITY B or FACILITY C**.

**Choice Set 1**

| **Attributes** | **FACILITY A** | **FACILITY B** | **FACILITY C** |
| --- | --- | --- | --- |
| **Waiting time before consultation** | 0 Less than 30mins | 1 30 mins – 1hr | 2 More than 1hr |
| **Information transfer from doctor to patient** | 0 Little or no information | 2 A lot of information | 1 Some information |
| **Availability of drugs and diagnostic equipment** | 0 Little or no drugs and diagnostic equipment | 1 Some drugs and diagnostic equipment | 2 A lot of drugs and diagnostic equipment |
| **Out-of-pocket costs per visit** | 0 #7,000 | 2 #13,000 | 1 #10,000 |
| **Pick your choice here**  **(please tick ONE box only)**🡪 |  |  |  |

**Choice Set 2**

| **Attributes** | **FACILITY A** | **FACILITY B** | **FACILITY C** |
| --- | --- | --- | --- |
| **Waiting time before consultation** | 0 Less than 30mins | 1 30mns- 1hr | 2 More than 1 hr |
| **Information transfer from doctor to patient** | 1 Some information | 0 Little or no information | 2 A lot of information |
| **Availability of drugs and diagnostic equipment** | 1 Some drugs and diagnostic equipment | 2 A lot of drugs and diagnostic equipment | 0 Little or no drugs and diagnostic equipment |
| **Out-of-pocket costs per visit** | 1 #10,000 | 0 #7,000 | 2 #13,000 |
| **Pick your choice here**  **(please tick ONE box only)**🡪 |  |  |  |

**Choice Set 3**

| **Attributes** | **FACILITY A** | **FACILITY B** | **FACILITY C** |
| --- | --- | --- | --- |
| **Waiting time before consultation** | 0 Less than 30mins | 1 30mins-1hr | 2 More than 1hr |
| **Information transfer from doctor to patient** | 2 A lot of information | 1 Some information | 0 Little or no information |
| **Availability of drugs and diagnostic equipment** | 2 A lot of drugs and diagnostic equipment | 0 Little or no drugs and diagnostic equipment | 1 Some drugs and diagnostic equipment |
| **Out-of-pocket costs per visit** | 2 #13,000 | 1 #10,000 | 0 #7,000 |
| **Pick your choice here**  **(please tick ONE box only)**🡪 |  |  |  |

**Choice Set 4**

| **Attributes** | **FACILITY A** | **FACILITY B** | **FACILITY C** |
| --- | --- | --- | --- |
| **Waiting time before consultation** | 1 30mins-1hr | 2 More than 1hr | 0 Less than 30mins |
| **Information transfer from doctor to patient** | 0 Little or no information | 2 A lot of information | 1 Some information |
| **Availability of drugs and diagnostic equipment** | 1 Some drugs and diagnostic equipment | 2 A lot of drugs and diagnostic equipment | 0 Little or no drugs and diagnostic equipment |
| **Out-of-pocket costs per visit** | 2 #13,000 | 1 #10,000 | 0 #7,000 |
| **Pick your choice here**  **(please tick ONE box only)**🡪 |  |  |  |

**Choice Set 5**

| **Attributes** | **FACILITY A** | **FACILITY B** | **FACILITY C** |
| --- | --- | --- | --- |
| **Waiting time before consultation** | 1 30mins-1hr | 2 More than 1hr | 0 Less than 30mins |
| **Information transfer from doctor to patient** | 1 Some information | 0 Little or no information | 2 A lot of information |
| **Availability of drugs and diagnostic equipment** | 2 A lot of drugs and diagnostic equipment | 0 Little or no drugs and diagnostic equipment | 1 Some drugs and diagnostic equipment |
| **Out-of-pocket costs per visit** | 0 #7,000 | 2 #13,000 | 1 #10,000 |
| **Pick your choice here**  **(please tick ONE box only)**🡪 |  |  |  |

**Choice Set 6**

| **Attributes** | **FACILITY A** | **FACILITY B** | **FACILITY C** |
| --- | --- | --- | --- |
| **Waiting time before consultation** | 1 30mins-1hr | 2 More than 1hr | 0 Less than 30mins |
| **Information transfer from doctor to patient** | 2 A lot of information | 1 Some information | 0 Little or no information |
| **Availability of drugs and diagnostic equipment** | 0 Little or no drugs and diagnostic equipment | 1 Some drugs and diagnostic equipment | 2 A lot of drugs and diagnostic equipment |
| **Out-of-pocket costs per visit** | 1 #10,000 | 0 #7,000 | 2 #13,000 |
| **Pick your choice here**  **(please tick ONE box only)**🡪 |  |  |  |

**Choice Set 7**

| **Attributes** | **FACILITY A** | **FACILITY B** | **FACILITY C** |
| --- | --- | --- | --- |
| **Waiting time before consultation** | 2 More than 1hr | 0 Less than 30mins | 1 30mins-1hr |
| **Information transfer from doctor to patient** | 0 Little or no information | 2 A lot of information | 1 Some information |
| **Availability of drugs and diagnostic equipment** | 2 A lot of drugs and diagnostic equipment | 0 Little or no drugs and diagnostic equipment | 1 Some drugs and diagnostic equipment |
| **Out-of-pocket costs per visit** | 1 #10,000 | 0 #7,000 | 2 #13,000 |
| **Pick your choice here**  **(please tick ONE box only)**🡪 |  |  |  |

**Choice Set 8**

| **Attributes** | **FACILITY A** | **FACILITY B** | **FACILITY C** |
| --- | --- | --- | --- |
| **Waiting time before consultation** | 2 More than 1hr | 0 Less than 30mins | 1 30mins-1hr |
| **Information transfer from doctor to patient** | 1 Some information | 0 Little or no information | 2 A lot of information |
| **Availability of drugs and diagnostic equipment** | 0 Little or no drugs and diagnostic equipment | 1 Some drugs and diagnostic equipment | 2 A lot of drugs and diagnostic equipment |
| **Out-of-pocket costs per visit** | 2 #13,000 | 1 #10,000 | 0 #7,000 |
| **Pick your choice here**  **(please tick ONE box only)**🡪 |  |  |  |

**Choice Set 9**

| **Attributes** | **FACILITY A** | **FACILITY B** | **FACILITY C** |
| --- | --- | --- | --- |
| **Waiting time before consultation** | 2 More than 1hr | 0 Less than 30mins | 1 30mins-1hr |
| **Information transfer from doctor to patient** | 2 A lot of information | 1 Some information | 0 Little or no information |
| **Availability of drugs and diagnostic equipment** | 1 Some drugs and diagnostic equipment | 2 A lot of drugs and diagnostic equipment | 0 Little or no drugs and diagnostic equipment |
| **Out-of-pocket costs per visit** | 0 #7,000 | 2 #13,000 | 1 #10,000 |
| **Pick your choice here**  **(please tick ONE box only)**🡪 |  |  |  |

**Choice Set 10**

| **Attributes** | **FACILITY A** | **FACILITY B** | **FACILITY C** |
| --- | --- | --- | --- |
| **Waiting time before consultation** | 0 Less than 30mins | 1 30mins-1hr | 2 More than 1hr |
| **Information transfer from doctor to patient** | 0 Little or no information | 2 A lot of information | 1 Some information |
| **Availability of drugs and diagnostic equipment** | 0 Little or no drugs and diagnostic equipment | 1 Some drugs and diagnostic equipment | 2 A lot of drugs and diagnostic equipment |
| **Out-of-pocket costs per visit** | 0 #7,000 | 2 #13,000 | 1 #10,000 |
| **Pick your choice here**  **(please tick ONE box only)**🡪 |  |  |  |
